# Estimating the Effects of Habitat and Biological Interactions in an Avian Community

**DOI:** 10.1371/journal.pone.0135987

**Published:** 2015-08-19

**Authors:** Robert M. Dorazio, Edward F. Connor, Robert A. Askins

**Affiliations:** 1 Southeast Ecological Science Center, U.S. Geological Survey, Gainesville, Florida, United States of America; 2 Department of Biology, San Francisco State University, San Francisco, California, United States of America; 3 Biology Department, Connecticut College, New London, Connecticut, United States of America; Università degli Studi di Milano-Bicocca, ITALY

## Abstract

We used repeated sightings of individual birds encountered in community-level surveys to investigate the relative roles of habitat and biological interactions in determining the distribution and abundance of each species. To analyze these data, we developed a multispecies N-mixture model that allowed estimation of both positive and negative correlations between abundances of different species while also estimating the effects of habitat and the effects of errors in detection of each species. Using a combination of single- and multispecies N-mixture modeling, we examined for each species whether our measures of habitat were sufficient to account for the variation in encounter histories of individual birds or whether other habitat variables or interactions with other species needed to be considered. In the community that we studied, habitat appeared to be more influential than biological interactions in determining the distribution and abundance of most avian species. Our results lend support to the hypothesis that abundances of forest specialists are negatively affected by forest fragmentation. Our results also suggest that many species were associated with particular types of vegetation as measured by structural attributes of the forests. The abundances of 6 of the 73 species observed in our study were strongly correlated. These species included large birds (American Crow (*Corvus brachyrhynchos*) and Red-winged Blackbird (*Agelaius phoeniceus*)) that forage on the ground in open habitats and small birds (Red-eyed Vireo (*Vireo olivaceus*), House Wren (*Troglodytes aedon*), Hooded Warbler (*Setophaga citrina*), and Prairie Warbler (*Setophaga discolor*)) that are associated with dense shrub cover. Species abundances were positively correlated within each size group and negatively correlated between groups. Except for the American Crow, which preys on eggs and nestlings of small song birds, none of the other 5 species is known to display direct interactions, so we suspect that the correlations may have been associated with species-specific responses to habitat components not adequately measured by our covariates.

## Introduction

Community ecology is largely driven by a desire to understand observed patterns of variation in the abundance or occurrence of species. In fact, one could argue that the holy grail of community ecology has been to understand the relative roles of the environment (habitat) and biological interactions in determining the distribution and abundance of species. This quest can be traced from early ideas about the processes driving succession and structuring plant communities [1–3], to debates about the roles of density-independent (driven by environment) and density-dependent (driven by species interactions) population regulation [4–6], to debates about the role of interspecific competition in structuring communities [7–9], and finally to alternative metacommunity theories about the formation and dynamics of spatially organized communities [10, 11].

Inferences about the effects of biological interactions and other potential drivers of community dynamics ideally should be based on a combination of observational data and experimental data, with the former being collected during surveys of the same locations over several years. However, longitudinal surveys and experiments involving entire communities are rare because it is difficult to sample entire communities at spatial scales that are relevant for inferring effects of habitat and biological interactions over a sustained period of time. More often, the conclusions of community ecologists are drawn from data collected during a single observational study, recognizing that attempts to infer the effects of ecological processes from a single ecological pattern are inherently limited.

Such observational studies often use presence-absence surveys, particularly in communities of high species richness. The data observed in these surveys can be analyzed using multispecies occupancy models [12–14], which account for errors in detection of species. These models of species occurrences are useful, but they are also limited. Occupancy state provides a relatively coarse summary of the abundance *N* of a species—specifically that *N* > 0 (species is present) or *N* = 0 (species is absent). The abundances of strongly interacting species are likely to be positively or negatively correlated, but these correlations can be difficult to detect because presence-absence surveys contain less information than abundance surveys. Community-level models have recently been proposed for estimating correlations in occurrence between species [15–19]; however, none of these models accounts for errors in detection, which is surprising because it is well known that presence-absence surveys of natural communities are prone to errors in detection [20–22]. At best, analyses of community-level data based on these newly proposed models may yield provisional estimates of the correlations between species; at worst, their estimates may contain substantial bias.

We believe that models of species-specific counts may be more useful for estimating the effects of biological interactions than models of presence-absence data. A variety of approaches have been used to analyze species-specific counts of individuals (plants or animals) encountered in community-level surveys. These approaches include linear-regression analysis, multivariate ordination, null-model analysis, Poisson-regression analysis, and N-mixture modeling (see [23] for a review). Only one of these approaches, N-mixture modeling, was designed to account for errors in detection of individuals. N-mixture models were proposed originally for the analysis of repeated point counts of a single species [24]. The counts observed at each sample location were modeled conditional on an unknown number *N* of individuals present at that location, and a second component of the model was used to specify how *N* varied among locations, often as a function of habitat measurements. N-mixture models have since been extended in many ways, particularly for the analysis of counts observed using other sampling protocols, such as double-observer, capture-recapture, or removal surveys [25]. More recently, N-mixture models have been developed for multiple species observed in community-level, point-count surveys [23, 26–28]. One of these models [23] allows abundances of different species to be correlated; however, the model is somewhat limited because correlations between abundances of different species were restricted to be positive and were specified as a function of interspecific differences in morphology. The ability to estimate the parameters of an unrestricted correlation matrix (containing positive or negative values) is obviously preferred because some interactions between species, such as competition, can produce negatively correlated abundances.

In this paper we used repeated sightings of individual birds encountered in community-level surveys to investigate the relative roles of habitat and biological interactions in determining the distribution and abundance of each species. Previous analyses of these surveys had used only the maximum number of birds detected per point-count survey [23, 29]. In the current paper we analyzed the entire encounter history of each bird detected in the community-level survey. We developed a multispecies N-mixture model of these encounter histories that allowed estimation of both positive and negative correlations between abundances of different species while also estimating the effects of habitat and the effects of errors in detection of each species. We do not claim that this model specifies or quantifies interactions between species mechanistically; it simply allows for covariation in abundances of different species. Potential sources of covariation include interactions between species and the effects of unobserved covariates, though neither of these sources can be distinguished unambiguously using observational data alone [18, 19, 30, 31]. Using a combination of single-species and multispecies N-mixture modeling, we examined for each species whether our measures of habitat were sufficient to account for the variation in encounter histories of individual birds or whether other habitat variables or interactions with other species needed to be considered. Thus, with respect to species interactions, our analysis was intended to be exploratory—that is, proposing hypotheses to explain covariance among different species—not confirmatory, where conclusive evidence for and against hypotheses is provided. Our analysis does indicate, however, that the vegetation and landscape variables frequently used to analyze bird communities was sufficient to explain the distributions of several species of birds without including the effects of biological interactions or other aspects of the habitat.

## Materials and Methods

### Study area and sampling methods

In 1983 and 1984 [29] sampled birds at 89 survey points (locations) in 46 forest patches in southeastern Connecticut. Forest patches were defined as regions of continuous forest separated from other forests by at least 10 m of non-forested habitat. The sizes of these patches ranged from 1.5 to 2600 ha and were scattered across a region whose area exceeded 2000 km^2^. Because the main goal of the original study was to investigate the effects of landscape variables on the distribution and abundance of bird species, sample locations were chosen to be relatively homogeneous in terms of vegetation structure and composition. All sites were located in closed-canopy upland broad-leaved forests, but analysis revealed substantial variation among sites in both landscape and vegetation characteristics.

To quantify variation among sites, measurements of 4 landscape and 11 vegetation variables were recorded at each site (S1 Dataset). The area of each forest patch, the amount of forest in the surrounding landscape, and the distance to the nearest forest patch were determined using USGS topographical maps and aerial photographs [29].

In previous studies [23, 29] statistical models were fitted to the maximum number of individuals (of each bird species) detected aurally (and sometimes visually) within a 100 m radius of the survey point. Repeated surveys of the same point were conducted during early morning hours (530 h to 1000 h) on three separate days during the breeding season (21 May to 11 July). In the present study we developed a model for the repeated sightings of each bird detected during a 20-minute survey. More specifically, during each survey an observer recorded the presence and location of each individual bird detected during each of four five-minute intervals. This was done primarily to help observers remain focused while tracking individual birds, but also with the hope that the resulting data might provide some insight into differences in detectability among species. In essence, this sampling protocol established a “capture history” of the detections and nondetections of each individual during the four five-minute intervals (borrowing a term used in analysis of mark-recapture data), though birds were never physically marked or handled in the surveys. Instead, major shifts in the position of each visible or vocalizing bird were continuously tracked with arrows on a map during the entire 20-minute observation period. If two or more individuals of the same species were detected simultaneously, we indicated this with pointer symbols, and then tracked each individual separately. One reason that we recorded positions for consecutive five-minute periods was to ensure that we repeatedly checked the position and the number of simultaneously singing individuals, and did not overlook additional conspecifics that had begun to sing. Most birds were detected because we heard their songs, and the tendency of territorial birds to respond repeatedly to the songs of conspecifics increased the effectiveness of simultaneous detections of different individuals.

Capture histories were recorded in 245 of the 267 surveys actually conducted (3 dates x 89 locations) because the method of recording birds in five-minute intervals was not adopted until after the first 22 surveys had been completed. We provide the data recorded during all 267 surveys (S1 Dataset).

The methods used in our surveys involved passive observation from fixed points with minimal disturbance to birds. No state or federal permits were required for this study because animals were not captured or collected. The surveys were completed in 1983 and 1984, before institutions were required to create an Institutional Animal Care and Review Committee (IACUC) under amendments to the Animal Welfare Act in 1985. Consequently, no review process for field studies of animals existed at Connecticut College at that time. Moreover, under current guidelines, observational studies of wild animals not collected, captured or manipulated do not require a review by the Connecticut College IACUC.

Our surveys were completed on 46 sites located within state-owned parks and forests, preserves owned by non-profit conservation groups, and private land. In each case we obtained permission to complete surveys from the land manager or owner. Some state-listed species were detected during surveys, but we were not required to have permits to observe or detect birds from a distance.

### Multispecies N-mixture model of correlated abundances

**Fig 1:**
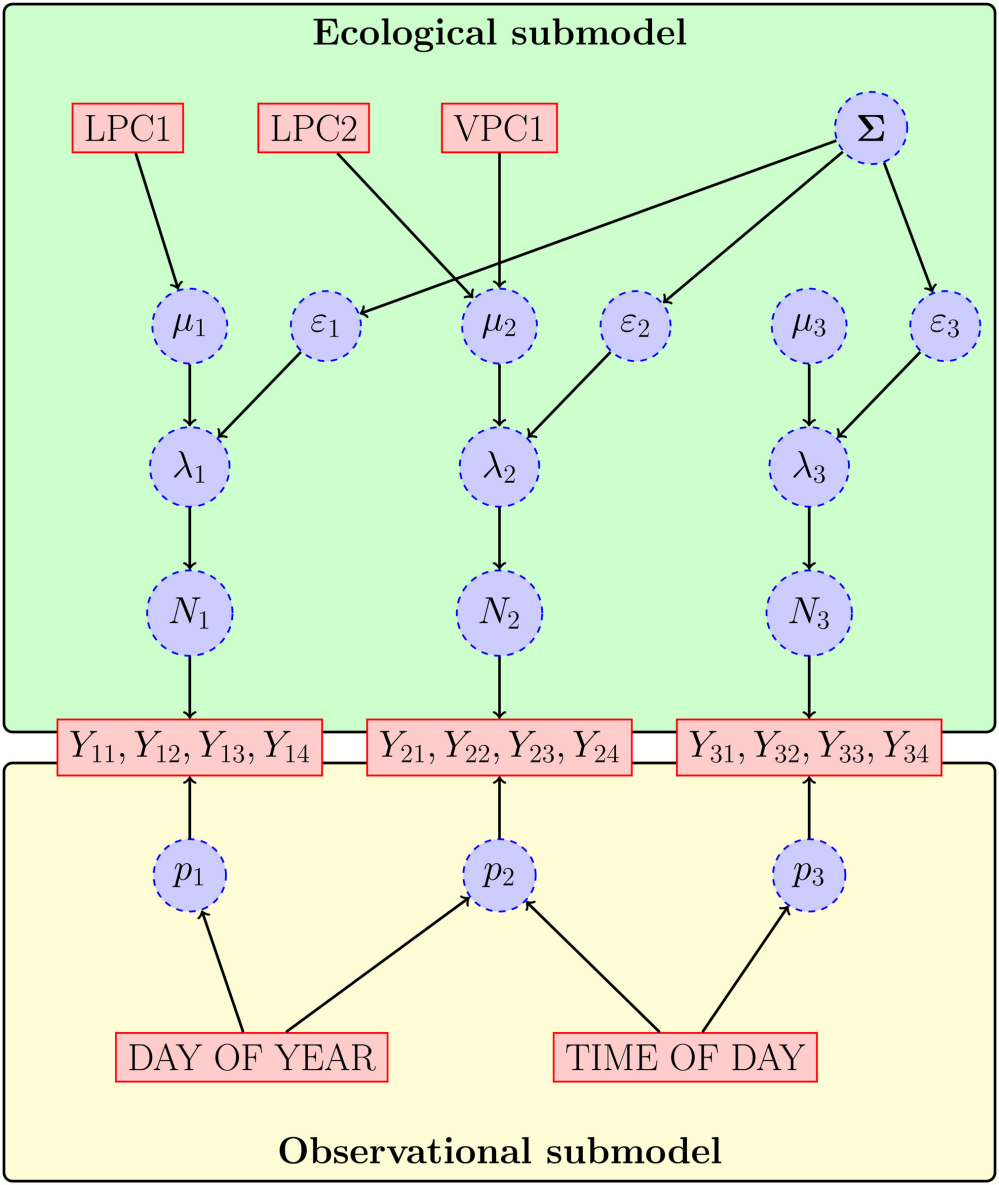
Schematic representation of the N-mixture model of correlated abundances for 3 species. Solid rectangles correspond to observed quantities (covariates or individual capture histories). Dashed circles correspond to latent random variables. Notation is described in Multispecies N-mixture model of correlated abundances. Covariates of abundance (LPC1, LPC2, VPC1) and detection probability (DAY OF YEAR, TIME OF DAY) are described in Statistical analysis of avian data.

In this section we describe a multispecies N-mixture model that includes two components: (1) an ecological submodel that specifies the effects of habitat and biological interactions on species-specific abundances, and (2) an observational submodel that specifies the effects of sampling, detection probabilities, and abundances on species-specific, individual encounter histories. Fig 1 provides a schematic representation of this model using covariates of abundance and detection that were observed in our surveys of forest birds.

#### Ecological submodel

In this section we describe a model of species-specific abundances, which are not directly observable but are the quantities of primary scientific interest. In the model we assume that each sample location is surveyed using the methods described in the previous section. In addition we assume that species abundances vary independently among surveys given a set of location- and survey-specific covariates (predictors of abundance). This assumption seems reasonable given the separation between sampled locations (which ranged from 0.25 to 61 km) and the maximum time between surveys of the same location (range: 12–36 days). During the breeding season movements of birds were limited, so they were unlikely to occupy more than one sample location during our surveys. However, the abundance or the number of birds available to be detected may have differed among surveys of the same location owing to species-specific nesting phenology. Our model allows the abundance of each species to differ among surveys of the same location, but it does not include parameters for temporal dependence that would be present in a dynamic model of nesting phenology.

Let ***N***_*i*_ = (*N*_*i*1_, …, *N*_*iK*_)′ denote a multivariate random variable composed of species-specific numbers of individuals that are present and available to be observed during the *i*th survey (*i* = 1, …, *I*). The number of distinct species (*K*) included in the analysis may equal all of the species observed in the sample or a set of focal species. Study objectives will usually determine the choice of *K*. We assume that ***N***_*i*_ has a multivariate Poisson-lognormal distribution [32], which we extend to include the effects of survey- and species-specific covariates as follows:

$$\begin{array}{ccc} \mathrm{Pr}(N_{i}=n_{i}|\varepsilon_{i}) & = & \prod_{k=1}^{K}f\left(n_{ik}|\exp\left(\mu_{ik}+\varepsilon_{ik}\right)\right) \end{array}$$ (1)

$$\begin{array}{ccc} \phi (\varepsilon_{i}) & = & {\left(2\pi \right)}^{-K/2}\;{\left|\Sigma \right|}^{-1/2}\exp\left(-0.5\;\varepsilon_{i}^{\prime }\Sigma^{-1}\varepsilon_{i}\right) \end{array}$$ (2)

where *f*(⋅∣*λ*_*ik*_) denotes the Poisson probability mass function with mean *λ*_*ik*_ = exp(*μ*_*ik*_ + *ɛ*_*ik*_) and where *ϕ*(**ε**_*i*_) denotes a *K*-variate normal (Gaussian) density of the residual vector **ε**_*i*_ = (*ɛ*_*i*1_, …, *ɛ*_*iK*_)′ with a mean vector of zeros and a *K* × *K* unrestricted covariance matrix **Σ** composed of variances $\sigma_{k}^{2}$ (diagonal elements) and covariances *ρ*_*kl*_ *σ*_*k*_ *σ*_*l*_ (off-diagonal elements); thus, **Σ** contains *K*(*K* + 1)/2 estimable parameters.

The multivariate Poisson-lognormal distribution is a mixture of Poisson distributions and allows us to specify the effects of habitat and biological interactions between species. For example, our extension of the multivariate Poisson-lognormal mixture includes a conditional, log-linear model for the expected abundance of individuals of species *k* present during survey *i*: log(*λ*_*ik*_) = *μ*_*ik*_ + *ɛ*_*ik*_. This model contains a linear predictor $\mu_{ik}=\beta_{k}^{\prime }x_{ik}$ and an error term *ɛ*_*ik*_. The linear predictor is composed of a vector of parameters ***β***_*k*_ and covariate measurements ***x***_*ik*_. Note that the expected abundance of each species is formulated as a function of survey- *and* species-specific covariates—that is, the same set of covariates is not necessarily used to model abundances of different species. This adds considerable flexibility to our multispecies N-mixture model that is not present in other models [23, 26–28] wherein abundances of all species are assumed to depend on the same set of covariates.

The residual error terms ***ε***_*i*_ (*i* = 1, …, *I*), which are assumed to be normally distributed, are used to specify the effects of unobserved (latent) sources of variability in species abundances. Although these sources of variability are unknown, they may correspond to the effects of unobserved habitat covariates or biological interactions between species (e.g., competitive interactions). To see this, consider a model of only *K* = 2 species. In this case the bivariate normal distribution of ***ε***_*i*_ implies the following conditional distributions:

$$\begin{array}{ccc} \varepsilon_{i1}|\varepsilon_{i2} & ∼ & \text{Normal}(\varepsilon_{i2}\frac{\rho_{12}\sigma_{1}}{\sigma_{2}},\;\sigma_{1}^{2}\left(1-\rho_{12}^{2}\right)) \end{array}$$ (3)

$$\begin{array}{ccc} \varepsilon_{i2}|\varepsilon_{i1} & ∼ & \text{Normal}(\varepsilon_{i1}\frac{\rho_{12}\sigma_{2}}{\sigma_{1}},\;\sigma_{2}^{2}\left(1-\rho_{12}^{2}\right)) \end{array}$$ (4)

In each of these distributions the conditional mean for the residual (log-scaled) abundance of one species depends on the residual abundance of the other species. Furthermore, both the direction (positive or negative) and magnitude of the association between species depend on the parameters in **Σ**. In fact, it is easily shown that this result generalizes to *K* species—that is, the conditional mean for the residual abundance of one species can be expressed as a linear combination of the residual abundances of all other species. An analyst may be tempted to interpret the coefficients of this linear combination strictly as the effects of biological interactions between species (e.g., “competition coefficients” [33–35]); however, in practice one cannot determine unambiguously whether the estimates of *ρ*_*kl*_ are associated with biological interactions or with unobserved habitat covariates [31]; therefore, care should be exercised in interpreting estimates of **Σ** and its parameters.

#### Observational submodel

We modeled the encounter histories of birds observed in each survey conditional on the latent number of birds present and available to be detected. In the *i*th survey individual birds were recorded as detected or not detected during each of *J*_*i*_ successive time intervals. Therefore, the data observed in each survey correspond to a set of “capture histories” for each individual bird.

We modeled the data for each species using a summary of the observed capture histories. Let ***Y***_*ik*_ = (*Y*_*ik*1_, …, *Y*_*ikJ*_*i*__)′ denote a random variable for the detection frequencies of birds of species *k* observed during the *i*th survey—that is, *Y*_*ikj*_ denotes a random variable for the number of individuals of species *k* detected exactly *j* times during the *i*th survey. To model these detection frequencies, we assume ***Y***_*ik*_ has a multinomial distribution, i.e.,

$$Y_{ik1},\ldots ,Y_{ikJ_{i}}|N_{ik}=n_{ik}∼\text{Multinomial}\left(n_{ik},\pi_{ik1},\ldots ,\pi_{ikJ_{i}}\right)$$ (5)

where πikj=(Jij)pikj(1−pik)Ji−j and *p*_*ik*_ is the probability of detecting a single individual of species *k* during an interval of observation associated with survey *i*.

We specify the effects of covariates on each species’ detection probability using the following logit-linear model: $\text{log}\{p_{ik}/(1-p_{ik})\}=\alpha_{k}^{\prime }w_{ik}$ where the linear predictor includes a vector of parameters ***α***_*k*_ and covariate measurements ***w***_*ik*_. Similar to the model of expected abundance, we specify *p*_*ik*_ as a function of survey- *and* species-specific covariates—that is, the same set of covariates is not necessarily used to model the detection probabilities of different species. We note, again, that this approach adds considerable flexibility to our multispecies N-mixture model.

### Statistical analysis of avian data

Our surveys were designed to sample an avian community of forest specialists that included both resident species and neotropical migrants (part-year residents). However, we also detected other (non-target) species capable of occupying forested and alternative habitats. In fact, some of these species were quite common and had established breeding territories in the vicinity of our sample locations. Rather than ignore these species, we decided to analyze the counts of every species detected in our surveys. This decision required us to develop an approach for distinguishing between species whose counts were adequately approximated by a single-species N-mixture model and species whose counts required the multispecies N-mixture model. This approach was necessary because the multispecies N-mixture model cannot be fitted to counts that lack overdispersion relative to a single-species N-mixture model (see Model selection for details). For now, we describe the potential covariates of abundance and detectability that were included in our analysis.

#### Abundance covariates

To specify differences in expected abundance of each species, we used measurements of 4 landscape covariates and 11 vegetation covariates (see Study area and sampling methods). Some of the measurements within each set of covariates were highly correlated. To establish a set of uncorrelated predictors of species abundances, we performed separate principal components analyses (PCAs) using the correlation matrices of the landscape- and vegetation-related measurements. The first two principal components of the landscape covariates (LPC1 and LPC2) accounted for 91.6% of the total variation in the measurements. LPC1 describes a gradient from small, isolated forests to large forests in heavily forested landscapes; thus, lower values of LPC1 correspond to higher levels of habitat fragmentation. The first six principal components of the vegetation covariates (VPC1–VPC6) accounted for 80% of the variation in the measurements. We therefore selected 8 principal components (LPC1, LPC2, and VPC1–VPC6) as potential regressors of expected abundances in the N-mixture models.

#### Detection covariates

We used the time of day (early, 5:30–7:00 h; middle, 7:00–8:30 h; or late, 8:30–10:00 h) and Julian day of year as potential covariates of detection probability of each species. Day of year is likely to be informative of detection because the frequency of singing of some species declined toward the end of the breeding period (late June through early July), so they were not as easy to detect.

#### Model selection

Our approach to model selection involved two steps. First, we fitted single-species N-mixture models to select the covariates of expected abundance and detection for each species. We chose the most parsimonious single-species model using the Bayesian information criterion (BIC). Second, if a single-species model failed to provide an adequate approximation of a species’ counts owing to overdispersion (as measured by the goodness-of-fit test described in S1 Appendix), the counts of that species were analyzed using the multispecies N-mixture model. This two-step procedure was needed because the multispecies N-mixture model cannot be fitted to counts that lack overdispersion relative to a single-species N-mixture model. To see this, note that marginalizing the multivariate Poisson-lognormal mixture yields the following expectations for *N*_*ik*_ and *N*_*il*_ (abundances of species *k* and *l* at survey point *i*):

$$\begin{array}{ccc} E(N_{ik}) & = & \tau_{ik} \end{array}$$ (6)

$$\begin{array}{ccc} \text{Var}(N_{ik}) & = & \tau_{ik}+\tau_{ik}^{2}\left\{\exp\left(\sigma_{k}^{2}\right)-1\right\} \end{array}$$ (7)

$$\begin{array}{ccc} \text{Cov}(N_{ik},N_{il}) & = & \tau_{ik}\tau_{il}\left\{\exp\left(\rho_{kl}\sigma_{k}\sigma_{l}\right)-1\right\} \end{array}$$ (8)

where $\tau_{ik}=\text{exp}(\mu_{ik}+\sigma_{k}^{2}/2)$ [32]. If there is no extra-Poisson variation in the abundance of species *k*, then *σ*_*k*_ = 0, E(*N*_*ik*_) = Var(*N*_*ik*_) = exp(*μ*_*ik*_), and Cov(*N*_*ik*_, *N*_*il*_) = 0. In other words, all of the variation in abundance of species *k* is expressed in the mean-variance relationship associated with the Poisson distribution. Furthermore, there is no covariance or correlation between the abundance of species *k* and the abundance of any other species.

Attempting to fit the multispecies N-mixture model to a species that lacks extra-Poisson variation in abundance is therefore doomed to failure. It may be possible to estimate the parameters of the Poisson mean *μ*_*ik*_, but the variance and covariance parameters (which equal zero) cannot be estimated for these species. Setting the variance and covariances to zero is also not an option because this produces a singular covariance matrix **Σ**, contradicting an assumption of the multispecies model. The two-step approach to model selection allowed us to identify the subset of species whose counts appeared to be overdispersed relative to a single-species N-mixture model (S1 Appendix).

#### Model fitting

Our multispecies N-mixture model would be difficult to fit using classical methods owing to the high-dimensional and analytically intractable integrations involved in evaluating the model’s marginal likelihood function. We therefore adopted a Bayesian approach to inference and used Markov chain Monte Carlo (MCMC) methods [36] to fit the model. In S2 Appendix we describe the MCMC algorithm used to fit our model and our choice of prior distributions for the model’s parameters. We provide the R code [37] used to fit the model and to compute posterior summaries of the model’s parameters in S1 Dataset.

## Results

Seventy three species of birds were detected at our survey locations (S1 Table). Based on our model selection procedure, the single species N-mixture model was appropriate for 57 of the 73 species encountered in our surveys. The observed counts of 28 of these species were best fit by an abundance model without habitat covariates (S3 Table). Fourteen of these species were detected in fewer than 5% of the surveys, and when present at most two individuals were detected (S2 Table). These species included forest specialists and species not normally associated with forested habitats. The other 14 species were detected in more surveys and in higher numbers, so the fact that they were best fit by a model without covariates suggests that our landscape and vegetation measurements were not informative measures of habitat for these species.

Of the 16 species for which the single species N-mixture model was not appropriate, the counts of 8 species could not be fitted to a multispecies N-mixture model because a maximum of only one bird was detected per survey and these detections occurred at relatively few survey locations (S2 Table). Counts of the remaining 8 species (Red-eyed Vireo (*Vireo olivaceus*), American Crow (*Corvus brachyrhynchos*), House Wren (*Troglodytes aedon*), Gray Catbird (*Dumetella carolinensis*), European Starling (*Sturnus vulgaris*), Hooded Warbler (*Setophaga citrina*), Prairie Warbler (*Setophaga discolor*), and Red-winged Blackbird (*Agelaius phoeniceus*)) were analyzed using the multispecies N-mixture model.

Most of the effect of landscape variables was mediated by LPC1, which describes a gradient from small, isolated forests to large forests in heavily forested landscapes. Among the species whose expected abundances increased with LPC1, ten are long-distance migrants that are forest specialists, one is a long distance migrant that is a young-forest specialist, and two are permanent residents associated with forest or young forest (S3 Table). In contrast, none of the 17 species whose expected abundances declined with LPC1 are forest or young-forest specialists. Six are early successional (shrubland) species, 10 are associated with open or edge habitats, and one is a generalist in edge and early successional habitats. Most of these species are short-distance migrants or permanent residents; only three species are long-distance migrants.

Among the species whose expected abundances were associated with vegetation, most were associated with either VPC1 or VPC2. VPC1 separates sample locations with mature forest from those with shrubby openings or an open canopy. Not surprisingly, the expected abundances of birds requiring a dense shrub layer (Yellow-billed Cuckoo (*Coccyzus americanus*), Common Yellowthroat (*Geothlypis trichas*), Eastern Towhee (*Pipilo erythrophthalmus*), and Black-and-white Warbler (*Mniotilta varia*)) increased with VPC1. The expected abundances of Red-eyed Vireo and Black-throated Green Warbler (*Setophaga virens*) declined with VPC1, indicating that they were more abundant at more mature sites. Among the species whose expected abundances were associated with VPC2, which separates sample locations with a higher proportion of conifers from those with more hardwoods and a denser, more diverse herb layer, most species were positively associated. Species with positive associations included ground feeders (Northern Bobwhite (*Colinus virginianus*), Veery (*Catharus fuscescens*), and Ovenbird (*Seiurus aurocapilla*)) as well as forest specialists (Broad-winged Hawk (*Buteo platypterus*), Brown Creeper (*Certhia americana*), Red-eyed Vireo, and Cerulean Warbler (*Setophaga cerulea*)), two early successional species (Blue-winged Warbler (*Vermivora cyanoptera*) and Common Yellowthroat (*Geothlypis trichas*)) and Red-bellied Woodpecker (*Melanerpes carolinus*). Hermit Thrush (*Catharus guttatus*) was the only species whose expected abundance declined with VPC2, indicating that it was more abundant at locations with more conifers.

The detection probabilities of seven species declined with day of year, indicating lower detectability later in the breeding season (S4 Table). These species are all long-distance migrants (Black-billed Cuckoo (*Coccyzus erythropthalmus*), Louisiana Waterthrush (*Parkesia motacilla*), Blue-winged Warbler, American Redstart (*Setophaga ruticilla*), Cerulean Warbler, and Chestnut-sided Warbler (*Setophaga pensylvanica*)) or an obligate brood parasite (Brown-headed Cowbird (*Molothrus ater*)). The detection probabilities of four species increased with day of year. These species (Hairy Woodpecker (*Picoides villosus*), American Crow, Black-capped Chickadee (*Poecile atricapillus*) and American Robin (*Turdus migratorius*)) are permanent residents.

Among the eight species analyzed with the multispecies N-mixture model, the abundances of two species (Gray Catbird and European Starling) were not significantly correlated with the abundances of any other species (Table 1). The abundances of the remaining six species, however, were strongly correlated. These species may be categorized into two groups: large species (American Crow and Red-winged Blackbird) that forage on the ground in open habitats, and small species (Red-eyed Vireo, House Wren, Hooded Warbler, and Prairie Warbler) that are associated with wooded or shrubby habitats. The abundances of American Crows and Red-winged Blackbirds were positively correlated with one another, and the abundances of all of the small species except Prairie Warbler were positively correlated with one another. The abundance of each large species was negatively correlated with the abundances of each small species (except for American Crow and Prairie Warbler).

**Table 1:** Estimated correlations between abundances of avian species fitted to the multispecies N-mixture model.

	Red-eyed Vireo	Hwa	Pw	Hwr	Gc	Rwb	Ac
Hooded Warbler (Hwa)	**0.74**						
	(0.006)						
Prairie Warbler (Pw)	0.55	**0.68**					
	(0.012)	(0.010)					
House Wren (Hwr)	**0.61**	**0.76**	**0.61**				
	(0.008)	(0.005)	(0.007)				
Gray Catbird (Gc)	0.03	0.07	-0.05	0.08			
	(0.019)	(0.021)	(0.020)	(0.019)			
Red-winged Blackbird (Rwb)	**-0.68**	**-0.77**	**-0.71**	**-0.69**	0.07		
	(0.008)	(0.006)	(0.009)	(0.006)	(0.020)		
American Crow (Ac)	**-0.58**	**-0.63**	-0.50	**-0.54**	-0.03	**0.63**	
	(0.009)	(0.010)	(0.014)	(0.010)	(0.018)	(0.009)	
European Starling	-0.17	-0.19	-0.41	-0.23	0.17	0.36	0.17
	(0.016)	(0.017)	(0.015)	(0.014)	(0.015)	(0.013)	(0.015)

Bold font indicates correlations that differed significantly from zero (using 5% significance level). Monte Carlo standard errors are given in parentheses.

## Discussion

Our results lend support to the hypothesis that abundances of forest specialists are negatively associated with the extent of forest fragmentation in the region around a survey point [38]. Abundances of ten of 17 species of forest-specialist, long-distance migrants were lower at higher levels of habitat fragmentation (as measured by lower values of LPC1). These results are consistent with other studies that reported evidence of negative responses of forest birds to habitat fragmentation [39]. The twelve species whose abundances were positively associated with habitat fragmentation are most abundant in non-forested habitats, such as open fields, early successional scrub or woodland edge [40]. It is therefore not surprising that these species were detected more frequently in less heavily forested landscapes.

Estimates of the effects of our four vegetation factors (VPC1–VPC4) suggest that many species were associated with a particular type of vegetation. Although all of the surveys were located in continuous, upland forest dominated by hardwood trees, these locations varied with respect to the diameter and height of the trees, the openness of the canopy, the density of conifers, tree diversity, and the density of shrubs and of herbs. For example, abundances of Red-eyed Vireo and Black-throated Green Warbler were higher at locations containing heavy tree canopy cover, whereas the abundances of several species (Yellow-billed Cuckoo, Common Yellowthroat and Eastern Towhee) that nest or forage in shrubs were higher at locations with a more open tree canopy. Many species were less abundant at sites with more conifers; only Hermit Thrush showed a positive association with conifers, which is consistent with descriptions of its habitat preferences [41].

The estimated effects of day of year on the detection probabilities of several species suggest that the seasonal timing of surveys is an important factor to consider when estimating abundance. Species whose detection probabilities were higher toward the end of the survey period are all permanent residents that usually begin nesting earlier in the spring than long-distance migrants. During late May (beginning of our surveys) these permanent residents tend to be incubating eggs and caring for young rather than singing or calling frequently. After their young fledge, the adults become more conspicuous as they move around in family groups and call frequently to their young. In contrast, the detection probabilities of seven species declined with day of year, and all but one of these species are long-distance migrants that usually set up territories and sing at a high frequency in May; the exception (Brown-headed Cowbird) is a brood parasite that often lays its eggs in the nests of long-distance migrants. By late June these migratory species become quieter as they care for eggs and nestlings.

In the forested region that we studied, habitat appeared to be more influential than biological interactions in determining the distribution and abundance of most avian species. Of the 65 species encountered frequently enough to fit models, 57 were best fitted by single-species N-mixture models while only 8 species had sufficient extra-Poisson variation to require a multispecies N-mixture model. Among the 57 species whose abundances were explained by single-species N-mixture models, the abundances of 28 species were adequately accounted for by the variance-mean relationship of the Poisson distribution without using habitat covariates. Hence, while habitat was influential in determining the abundances of the majority of species, a substantial number required neither habitat nor biological interactions to account for the variation in their abundances.

Of the 8 species for which a multispecies N-mixture model was required, we found significant correlations in abundance among only 6 species. Of 13 significant correlations, 7 were negative and could conceivably be interpreted as evidence of interspecific competition. The 6 positive correlations could conceivably be interpreted as evidence of heterospecific attraction [42, 43]. However, as described earlier (see Multispecies N-mixture model of correlated abundances), we cannot be certain if these correlations were associated with biological interactions or with unobserved environmental covariates. The American Crow preys on the eggs and nestlings of smaller songbirds and may have an impact on reproductive rates and density along forest edges where crows are common. Three of the four species that show a negative relationship with crows (House Wren, Hooded Warbler and Prairie Warbler) are shrub-layer species that are associated with canopy gaps of open forest. Perhaps these species are more susceptible to nest predation by crows because of the open tree canopy. For the remainder of these species, none are known to display obvious direct interspecific interactions, such as predation, brood parasitism, competition for food or nest sites, or participation in the same mixed species flocks (heterospecific attraction). Instead, the correlations between species may reflect responses to components of habitat not adequately measured by our landcape and vegetation covariates. We suspect that the positive correlation between abundances of American Crows and Red-winged Blackbirds reflects similar habitat requirements; both species are large omnivores that forage on the ground in open habitats. Furthermore, their abundances were negatively correlated with those of the three species of small insectivores (Red-eyed Vireo, House Wren, and Hooded Warbler) that are not found in open habitats. The abundances of all four small species were positively correlated with one another except for Prairie Warbler with Red-eyed Vireo. Prairie Warblers are associated with open shrublands while Red-eyed Vireos are primarily found in forests [40]. House Wren, Hooded Warbler and Prairie Warbler are all associated with dense shrub cover [40], and Red-eyed Vireo is described as being scarce at sites with little or no shrub cover [44]; therefore, the use of shrub cover may be a common denominator for these four species even though our analysis indicated that abundance of Red-eyed Vireo was higher in mature forests with relatively little shrub cover. It is possible that the distribution of these species is influenced by features of the shrub layer that we did not measure (such as prey density or plant species composition of the shrub layer), but this is conjecture on our part. Additional research is needed to clarify the specific habitat requirements of these species.

Quests for evidence of competitive interactions often attempt to identify negative associations between guild members, closely related species, or species with similar morphology. However, in our study 3 of the 6 positive correlations were among guild members (Prairie Warbler, Hooded Warbler, and House Wren [45]), and the only correlation among congeneric species also was positive (Prairie Warbler and Hooded Warbler). Furthermore, these positive correlations appeared to be unrelated to the measure of morphological dissimilarity used by [23]. All of the negative correlations that we estimated were between species that are in different guilds, different genera, and are remarkably different in morphology. So, even if future studies are unable to discover environmental covariates sufficient to account for the observed negative correlations, competition would remain an unlikely explanation.

The spatial extent of our point count surveys is arguably an appropriate scale with which to estimate avian abundances during the breeding season because territorial interactions and habitat preferences occur at this scale. We do not mean to preclude the possibility that evidence of interactions may also occur at larger spatial scales or that the environment as manifested by regional differences in climate and topography also may influence abundances at larger scales. However, even species turnover at larger scales cannot be attributed solely to biological interactions or to the environment [18, 19]. The ambiguity of our interpretation is not a characteristic of the models we fitted, but rather a trait of the observational data on which they are based. Yet, even at the scale of our study experimental manipulations would be prohibitive if not prohibited. Models that can estimate species abundances while accounting for imperfect detection, the effects of the environment, and associations between species are a substantial improvement over those to which we have been limited during the past 40 years. Furthermore, our approach to model selection allowed us to distinguish species for which associations among species are useful in modeling abundances from species which may be treated as if biological interactions are unimportant. We found that for most species (57 of 65 for which models could be fitted) associations among species were not required to model their abundances.

We developed the multispecies N-mixture model to provide a useful starting point for the analysis of data observed in community-level surveys of birds and other taxa. The model cannot be used to determine the underlying causes of correlation between abundances of different species; however, by combining correlation estimates with detailed knowledge of the ecology and behaviors of individual species—as was done in our analysis of avian species—a firmer basis for ecological inferences can be established.

The multispecies N-mixture model that we developed can be extended in several ways. Spatial random effects can be added to specify the effects of unobserved habitat covariates on the spatial distribution of abundance of each species [46, 47]. If these effects were important, this extension could reduce estimates of correlation between abundances of non-interacting species. The multispecies N-mixture model also can be extended to model the dynamics of communities monitored over time. For example, if longitudinal surveys of communities were available, a spatially explicit extension of the model that included dispersal of individuals could be used to provide a framework for comparing alternative metacommunity theories. Finally, the observation component of our model could easily be revised for data produced by alternative sampling protocols, such as double-observer or removal surveys, that induce a multinomial distribution of counts.

## Supporting Information

S1 Dataset Data sets and R code. Data are stored in three files: habitat, species list codes, and observations of individual birds. The file AnalysisOfAskinsMultinomCountsByMLE.R contains R code for fitting single-species N-mixture models. The file AnalysisOfAskinsMultinomCounts.R contains R code for fitting multi-species N-mixture models.
(ZIP)

S1 Appendix Model selection procedure. Description of procedure used to select covariates of abundance and detection for each species and to assess the selected N-mixture model’s goodness of fit.
(PDF)

S2 Appendix MCMC algorithm used to fit multispecies N-mixture model. Description of technical details of fitting the multispecies N-mixture model.
(PDF)

S1 Table List of species. Common name, scientific name, and primary habitat of each species.
(PDF)

S2 Table Summary of avian counts by species. (PDF)

S3 Table Species-specific estimates of abundance parameters. (PDF)

S4 Table Species-specific estimates of detection parameters. (PDF)
