# Supplementary material for: Estimating the Effects of Habitat and Biological Interactions in an Avian Community
Source: PLoS One. 2015 Aug 19;10(8):e0135987. doi: 10.1371/journal.pone.0135987 (PMC4543583; doi:10.1371/journal.pone.0135987)
Supplement: S1 Appendix — Description of procedure used to select covariates of abundance and detection for each species and to assess the selected N-mixture model’s goodness of fit. (PDF) [file pone.0135987.s002.pdf]

Estimating the effects of habitat and biological interactions in an avian community

Robert M. Dorazio, U.S. Geological Survey, Southeast Ecological Science Center, Gainesville, FL, USA

Edward F. Connor, Department of Biology, San Francisco State University, San Francisco, CA, USA

Robert A. Askins, Biology Department, Connecticut College, New London, CT USA

S1 Appendix: Model selection procedure

Covariate selection

For each species we selected covariates of expected abundance first and then chose the detection covariates conditional on the subset of selected abundance covariates. This approach seemed sensible for two reasons: (1) the potential covariates of expected abundance were distinct from those of detection probability and (2) most of the variation in species abundances among survey locations was thought – a priori – to reflect differences in the values of habitat (landscape and vegetation) covariates. Therefore, in the first step of covariate selection, we used Poisson regression models wherein the total number of detected individuals in a survey was used as a response for each species. In other words, we treated the total number of detected individuals in a survey as a surrogate for the true, but unknown, abundance of a species. From all possible combinations of the 8 potential covariates of abundance (LPC1, LPC2, and VPC1–VPC6), we selected those covariates with the lowest value of BIC. This selection procedure yielded either 0, 1, or 2 covariates of expected abundance depending on the species. Covariates VPC5 and VPC6 were not selected for any species, and covariates VPC3 and VPC4 were selected for relatively few species.

To select the detection covariates for each species, we fitted single-species N-mixture models that may be regarded as a restricted version of our multispecies N-mixture model

wherein $\varepsilon_{ik} = 0$ is assumed for all species and surveys. In other words, each single-species model is a multinomial-Poisson mixture:

$$N_{ik} \sim \text{Poisson}(\exp(\mu_{ik})) \quad (1)$$

$$Y_{ik1}, \ldots, Y_{ikJ_i} | N_{ik} = n_{ik} \sim \text{Multinomial}(n_{ik}, \pi_{ik1}, \ldots, \pi_{ikJ_i}) \quad (2)$$

Recall that the effects of detection covariates are specified within each multinomial probability π_{ikj}. Using the method of maximum likelihood, we fitted this class of models for all possible combinations of detection covariates and then selected a specific set of detection covariates from the N-mixture model with the lowest value of BIC. Depending on species, this selection procedure yielded either no covariate or a single detection covariate (day of year). Time of day was not selected as a detection covariate for any species.

Species selection

We also developed a procedure for assessing whether the single-species N-mixture model (described earlier) provided an adequate approximation of the counts of each species. This procedure essentially provides a goodness-of-fit test for the N-mixture model. If the test suggests lack of fit, we may conclude that the counts were overdispersed relative to the single-species model. In this case the multispecies N-mixture model would be more appropriate for analyzing the counts of that species.

Under the assumptions of the single-species N-mixture model (Equations 1 and 2), it is easily shown that

$$\Pr(Y_{ik1} = y_{ik1}, \ldots, Y_{ikJ_i} = y_{ikJ_i}) = \prod_{j=1}^{J_i} \frac{\exp\{-\pi_{ikj} \exp(\mu_{ik})\}\{\pi_{ikj} \exp(\mu_{ik})\}^{y_{ikj}}}{y_{ikj}!}$$

In other words, each survey's counts are independent and have a Poisson distribution: $Y_{ikj} \sim \text{Poisson}(\pi_{ikj} \exp(\mu_{ik}))$. A sum of independent Poisson random variables also has a Poisson

distribution; therefore, for our model $Y_{ik\cdot} \sim \text{Poisson}(\pi_{ik\cdot} \exp(\mu_{ik}))$ where $Y_{ik\cdot} = \sum_{j=1}^{J_i} Y_{ikj}$ is a random variable for the total number of birds of species k detected during the ith survey and where $\pi_{ik\cdot} = \sum_{j=1}^{J_i} \pi_{ikj}$.

For each species, the I values of $Y_{ik\cdot}$ are independent given our sampling design and modeling assumptions. Therefore, the deviance statistic for Poisson models [1]

$$D = 2\sum_{i=1}^{I} y_{ik\cdot} \log\left\{\frac{y_{ik\cdot}}{\hat{\pi}_{ik\cdot} \exp(\hat{\mu}_{ik})}\right\} - y_{ik\cdot} + \hat{\pi}_{ik\cdot} \exp(\hat{\mu}_{ik})$$

provides a measure of the discrepancy between the total number of birds observed in a survey ($y_{ik\cdot}$) and the total number of birds expected in a survey ($\hat{\pi}_{ik\cdot} \exp(\hat{\mu}_{ik})$) given the assumptions of the single-species N-mixture model and the estimates of its parameters. (We use circumflexes to indicate quantities computed from parameter estimates.) Unlike simple Poisson models, the sampling distribution of D is not generally known, so we used parametric bootstrapping to approximate this distribution.

For each species we assessed goodness of fit by estimating the p-value associated with the observed value D_{obs} of the deviance test statistic. We estimated $p = \Pr(D > D_{obs})$ from a parametrically bootstrapped sample of M values of D by assuming that the random variable $X = I(D > D_{obs})$ has a Bernoulli distribution with success parameter p. The maximum likelihood estimator of the p-value therefore equals $\hat{p} = (1/M)\sum_{i=1}^{M} x_i$, and an asymptotically valid 95% confidence interval for p may be computed as follows: $\hat{p} \pm 1.96\sqrt{\hat{p}(1-\hat{p})/M}$. As a hedge against the chance that our goodness-of-fit test lacked power, we assumed a significance level of $\alpha = 0.10$. In addition, we used the lower confidence limit for p to assess lack of fit; thus, we concluded that the single-species N-mixture model did not adequately approximate the counts of a species if $\hat{p} - 1.96\sqrt{\hat{p}(1-\hat{p})/M} < \alpha$.

References

[1] Alan Agresti. *Categorical Data Analysis, second edition*. Wiley, Hoboken, New Jersey, 2002.
