# Supplementary material for: Estimating the Effects of Habitat and Biological Interactions in an Avian Community
Source: PLoS One. 2015 Aug 19;10(8):e0135987. doi: 10.1371/journal.pone.0135987 (PMC4543583; doi:10.1371/journal.pone.0135987)
Supplement: S2 Appendix — Description of technical details of fitting the multispecies N-mixture model. (PDF) [file pone.0135987.s003.pdf]

Estimating the effects of habitat and biological interactions in an avian community

Robert M. Dorazio, U.S. Geological Survey, Southeast Ecological Science Center, Gainesville, FL, USA

Edward F. Connor, Department of Biology, San Francisco State University, San Francisco, CA, USA

Robert A. Askins, Biology Department, Connecticut College, New London, CT USA

S2 Appendix: MCMC algorithm used to fit multispecies N-mixture models

In this supplement we describe the MCMC algorithm that was used to compute summaries of the posterior distribution of model parameters. We use bracket notation [1] to specify probability density functions; thus, $[x, y]$ denotes the joint density of random variables X and Y, $[x|y]$ denotes the conditional density of X given $Y = y$, and $[x]$ denotes the unconditional (marginal) density of X.

We used the MCMC algorithm to generate a Markov chain whose stationary distribution is equivalent to a posterior with density function proportional to

$$[\boldsymbol{\beta}, \boldsymbol{\alpha}, \boldsymbol{\Sigma}^{-1}, \boldsymbol{b}, \boldsymbol{\varepsilon}, \boldsymbol{n}|\boldsymbol{y}] = [\boldsymbol{\beta}][\boldsymbol{\alpha}][\boldsymbol{b}][\boldsymbol{\Sigma}^{-1}|\boldsymbol{b}] \prod_{i=1}^{I}[\boldsymbol{\varepsilon}_i|\boldsymbol{\Sigma}^{-1}] \prod_{k=1}^{K}[n_{ik}|\lambda_{ik}][\boldsymbol{y}_{ik}|n_{ik}, p_{ik}]$$

where $\boldsymbol{\beta} = (\boldsymbol{\beta}_1, \ldots, \boldsymbol{\beta}_K)$, $\boldsymbol{\alpha} = (\boldsymbol{\alpha}_1, \ldots, \boldsymbol{\alpha}_K)$, $\boldsymbol{\varepsilon} = (\boldsymbol{\varepsilon}_1, \ldots, \boldsymbol{\varepsilon}_I)$, and $\boldsymbol{n} = (\boldsymbol{n}_1, \ldots, \boldsymbol{n}_I)$ are parameters of the multispecies N-mixture model. The vector $\boldsymbol{b} = (b_1, \ldots, b_K)'$ contains auxiliary parameters that were used to specify the prior distribution of $\boldsymbol{\Sigma}$ hierarchically [2] as described below. The vector $\boldsymbol{y}_{ik} = (y_{ik1}, \ldots, y_{ikJ_i})'$ contains the detection frequencies of birds of species k observed during the ith survey, and $\boldsymbol{y} = \{\boldsymbol{y}_{ik}, \forall ik\}$ denotes the entire set of observations.

The MCMC algorithm uses either a Gibbs or a Metropolis-Hastings (M-H) sampler depending on the parameter. Each of the following full-conditional distributions is sampled in one iteration of the algorithm:

1. The full conditional for N_{ik} has a familiar form: $N_{ik} - y_{ik\cdot}|\cdot \sim \text{Poisson}(\lambda_{ik}(1-p_{ik})^{J_i})$ where $y_{ik\cdot} = \sum_{j=1}^{J_i} y_{ikj}$ is the total number of birds of species k detected during the ith survey. In other words, this is the conditional distribution of the number of birds that were present but not detected during the survey.

2. We assumed a hierarchical prior distribution for $\mathbf{\Sigma}$ [2] that allowed us to specify marginally noninformative priors for its elements. Specifically, the hyperparameters of this prior can be chosen so that each standard deviation parameter σ_k has a Half-t prior of arbitrarily high noninformity [3] and each correlation parameter ρ_{kl} has a uniform prior on (-1,1). The hierarchical prior distribution for $\mathbf{\Sigma}$ is a mixture of Inverse-Wishart and Inverse-Gamma distributions:

$$\begin{aligned} \mathbf{\Sigma}^{-1}|b_1,\ldots,b_K &\sim \text{Wishart}(\nu+K-1,\ (2\nu\boldsymbol{B})^{-1}) \\ b_k &\sim \text{Gamma}(1/2, 1/s_k^2) \end{aligned}$$

   where $\boldsymbol{B} = \text{diag}(b_1,\ldots,b_K)$. [2] showed that the marginal prior density for ρ_{kl} is proportional to $(1-\rho_{kl})^{\nu/2-1}$; therefore, we let $\nu = 2$ to specify a marginally uniform prior for each correlation parameter. [2] also showed that the marginal prior distribution for σ_k is a Half-t distribution with ν degrees of freedom and scale parameter s_k; therefore, we specified a noninformative prior for σ_k by choosing s_k to be arbitrarily high.

   The conditional conjugacy of this prior for $\mathbf{\Sigma}$ leads to full conditional distributions of familiar form that are relatively easy to sample. Specifically,

$$\mathbf{\Sigma}^{-1}|\cdot \sim \text{Wishart}(\nu+I+K-1,\ (2\nu\boldsymbol{B}+\boldsymbol{E}'\boldsymbol{E})^{-1})$$

where the $I \times K$ matrix $\boldsymbol{E} = (\boldsymbol{\varepsilon}_1, \boldsymbol{\varepsilon}_2, \ldots, \boldsymbol{\varepsilon}_I)'$, and

$$b_k \sim \text{Gamma}((\nu + K)/2,\ 1/s_k^2 + \nu(\boldsymbol{\Sigma}^{-1})_{kk})$$

where $(\boldsymbol{\Sigma}^{-1})_{kk}$ is the kth diagonal element of $\boldsymbol{\Sigma}^{-1}$. Once $\boldsymbol{\Sigma}^{-1}$ is drawn from its full conditional, it is a simple matter to compute the matrix of correlation parameters as follows: $\{\text{diag}(\boldsymbol{\Sigma})\}^{-1/2}\boldsymbol{\Sigma}\{\text{diag}(\boldsymbol{\Sigma})\}^{-1/2}$.

3. The full conditional density of $\boldsymbol{\varepsilon}$ factors into a product of I independent terms. To sample the ith target density, which is proportional to

$$\begin{aligned} [\boldsymbol{\varepsilon}_i|\cdot] &= [\boldsymbol{\varepsilon}_i|\boldsymbol{\Sigma}^{-1}] \prod_{k=1}^{K} [n_{ik}|\lambda_{ik}] \\ &= \exp\left[-\boldsymbol{\varepsilon}_i'\boldsymbol{\Sigma}^{-1}\boldsymbol{\varepsilon}_i + \sum_{k=1}^{K} n_{ik}\log(\lambda_{ik}) - \lambda_{ik}\right] \end{aligned}$$

we used the M-H algorithm. Following [4], we used a multivariate-t distribution as a proposal and selected its parameters to approximate the target distribution. Specifically, let $f(\boldsymbol{\varepsilon}_i) = \log([\boldsymbol{\varepsilon}_i|\cdot])$. We assigned the mean of the proposal distribution to equal $\hat{\boldsymbol{\varepsilon}}_i$, the value of $\boldsymbol{\varepsilon}_i$ that maximized $f(\boldsymbol{\varepsilon}_i)$. This maximimization was done numerically using an analytical gradient $\boldsymbol{g}(\boldsymbol{\varepsilon}_i) = -\boldsymbol{\Sigma}^{-1}\boldsymbol{\varepsilon}_i + \boldsymbol{n}_i - \boldsymbol{\lambda}_i$ and hessian $\boldsymbol{H}(\boldsymbol{\varepsilon}_i) = -\boldsymbol{\Sigma}^{-1} - \text{diag}(\boldsymbol{\lambda}_i)$. The covariance of the proposal distribution was then computed by inverting the negative of the hessian matrix $\{-\boldsymbol{H}(\hat{\boldsymbol{\varepsilon}}_i)\}^{-1}$. The degrees of freedom parameter of the proposal may be used as a tuning parameter; however, in practice we simply assigned this parameter to be constant (2.0).

4. The full conditional density of $\boldsymbol{\beta}$ factors into a product of K independent terms. For each term we assumed a normal prior distribution with mean zero and diagonal covariance matrix $\boldsymbol{V}_{\beta_k}$, using an arbitrarily high value for the nonzero elements of $\boldsymbol{V}_{\beta_k}$

to specify prior ignorance. To sample the kth target density, which is proportional to

$$\begin{aligned}[\boldsymbol{\beta}_k|\cdot] &= [\boldsymbol{\beta}_k]\prod_{i=1}^{I}[n_{ik}|\lambda_{ik}] \\ &= \exp\left[-\boldsymbol{\beta}_k'\boldsymbol{V}_{\beta_k}^{-1}\boldsymbol{\beta}_k + \sum_{i=1}^{I} n_{ik}\log(\lambda_{ik}) - \lambda_{ik}\right]\end{aligned}$$

we used the M-H algorithm. Following [4], we used a multivariate-t distribution as a proposal and selected its parameters to approximate the target distribution. Specifically, let $f(\boldsymbol{\beta}_k) = \log([\boldsymbol{\beta}_k|\cdot])$. We assigned the mean of the proposal distribution to equal $\hat{\boldsymbol{\beta}}_k$, the value of $\boldsymbol{\beta}_k$ that maximized $f(\boldsymbol{\beta}_k)$. This maximimization was done numerically, and the covariance of the proposal distribution was then computed by inverting the negative of the hessian matrix $\{-\boldsymbol{H}(\hat{\boldsymbol{\beta}}_k)\}^{-1}$. The degrees of freedom parameter of the proposal may be used as a tuning parameter; however, in practice we simply assigned this parameter to be constant (2.0).

5. The full conditional density of $\boldsymbol{\alpha}$ factors into a product of K independent terms. Let Q_k equal the number of parameters in $\boldsymbol{\alpha}_k$. We assumed a noninformative prior for $\boldsymbol{\alpha}_k$ that comprises Q_k independent t-distributions, each with mean zero, scale parameter $\sigma_\alpha = 1.566$, and degrees of freedom parameter $\nu_\alpha = 7.763$. This distribution approximates a Uniform$(0,1)$ prior on the inverse-logit scale and assigns low probabilities to values outside the interval (-5,5) [5]. To sample the kth target density, which is proportional to

$$\begin{aligned}[\boldsymbol{\alpha}_k|\cdot] &= [\boldsymbol{\alpha}_k]\prod_{i=1}^{I}[\boldsymbol{y}_{ik}|n_{ik}, p_{ik}] \\ &= \exp\left[\sum_{q=1}^{Q_k} -\frac{\nu_\alpha+1}{2}\log\left\{1+\frac{\alpha_{kp}^2}{\nu_\alpha\sigma_\alpha^2}\right\} + \sum_{i=1}^{I}(n_{ik}-y_{ik\cdot})\log(\pi_{ik0}) + \sum_{j=1}^{J_i} y_{ikj}\log(\pi_{ikj})\right]\end{aligned}$$

we used the M-H algorithm. We used a multivariate-t distribution as a proposal and selected its parameters to approximate the target distribution. Specifically, let $f(\boldsymbol{\alpha}_k) =$

$\log([\boldsymbol{\alpha}_k|\cdot])$. We assigned the mean of the proposal distribution to equal $\hat{\boldsymbol{\alpha}}_k$, the value of $\boldsymbol{\alpha}_k$ that maximized $f(\boldsymbol{\alpha}_k)$. This maximimization was done numerically, and the covariance of the proposal distribution was then computed by inverting the negative of the hessian matrix $\{-\boldsymbol{H}(\hat{\boldsymbol{\alpha}}_k)\}^{-1}$. The degrees of freedom parameter of the proposal may be used as a tuning parameter; however, in practice we simply assigned this parameter to be constant (2.0).

We used $M = 150000$ iterations of the MCMC algorithm to estimate summaries (means, standard deviations, quantiles) of the posterior distribution and other ecologically relevant functionals of the Markov chain. The estimates were computed using ergodic averages, which are simulation consistent (that is, the averages converge to posterior expectations as the number of iterations increases) according to the strong law of large numbers for Markov chains [6]. (The first 20000 elements of the Markov chain were not used because this part of the chain appeared to be transient.) Monte Carlo standard errors of the estimates were computed using the subsampling bootstrap method [7, 6] with overlapping batch means of size $\lfloor\sqrt{M}\rfloor$.

References

[1] A. E. Gelfand and A. F. M. Smith. Sampling-based approaches to calculating marginal densities. *Journal of the American Statistical Association*, 85:398–409, 1990.

[2] A. Huang and M. P. Wand. Simple marginally noninformative prior distributions for covariance matrices. *Bayesian Analysis*, 8:439–452, 2013.

[3] A. Gelman. Prior distributions for variance parameters in hierarchical models (Comment on article by Browne and Draper). *Bayesian Analysis*, 1:515–534, 2006.

[4] S. Chib and R. Winkelmann. Markov chain Monte Carlo analysis of correlated count data. *Journal of Business & Economic Statistics*, 19:428–435, 2001.

[5] R. M. Dorazio, N. J. Gotelli, and A. M. Ellison. Modern methods of estimating biodiversity from presence-absence surveys. In O. Grillo and G. Venora, editors, *Biodiversity Loss in a Changing Planet*, pages 277–302. InTech, Rijeka, Croatia, 2011.

[6] J. M. Flegal and G. L. Jones. Implementing MCMC: estimating with confidence. In S. Brooks, A. Gelman, G. L. Jones, and X. L. Meng, editors, *Handbook of Markov chain Monte Carlo*, pages 175–197. Chapman & Hall / CRC, Boca Raton, Florida, 2011.

[7] J. M. Flegal and G. L. Jones. Batch means and spectral variance estimators in Markov chain Monte Carlo. *Annals of Statistics*, 38:1034–1070, 2010.
