# Supplementary material for: Estimating the Effects of Habitat and Biological Interactions in an Avian Community
Source: PLoS One. 2015 Aug 19;10(8):e0135987. doi: 10.1371/journal.pone.0135987 (PMC4543583; doi:10.1371/journal.pone.0135987)
Supplement: S1 Table — Common name, scientific name, and primary habitat of each species. (PDF) [file pone.0135987.s004.pdf]

Estimating the effects of habitat and biological interactions in an avian community

Robert M. Dorazio, U.S. Geological Survey, Southeast Ecological Science Center, Gainesville, FL, USA

Edward F. Connor, Department of Biology, San Francisco State University, San Francisco, CA, USA

Robert A. Askins, Biology Department, Connecticut College, New London, CT USA

S1 Table: List of species

List of species (with habitat) observed at survey locations. Habitat categories are based on detailed descriptions of habitat use of New England bird species published by [1].

Scientific name	Common name	Habitat
Long-distance migrants		
Buteo platypterus	Broad-winged Hawk	forest
Coccyzus americanus	Yellow-billed Cuckoo	early succession
Coccyzus erythropthalmus	Black-billed Cuckoo	early succession
Antrostomus vociferus	Eastern Whip-poor-will	young forest
Archilochus colubris	Ruby-throated Hummingbird	early succession
Contopus virens	Eastern Wood-Pewee	forest
Empidonax virescens	Acadian Flycatcher	forest
Myiarchus crinitus	Great Crested Flycatcher	forest
Tyrannus tyrannus	Eastern Kingbird	open
Vireo flavifrons	Yellow-throated Vireo	forest
Vireo olivaceus	Red-eyed Vireo	forest
Polioptila caerulea	Blue-gray Gnatcatcher	forest

Continued on next page

Scientific name	Common name	Habitat
Catharus fuscescens	Veery	young forest
Catharus guttatus	Hermit Thrush	forest
Hylocichla mustelina	Wood Thrush	forest
Seiurus aurocapilla	Ovenbird	forest
Helmitheros vermivorum	Worm-eating Warbler	forest
Parkesia motacilla	Louisiana Waterthrush	forest
Vermivora cyanoptera	Blue-winged Warbler	early succession
Mniotilta varia	Black-and-white Warbler	forest
Setophaga citrina	Hooded Warbler	young forest
Setophaga ruticilla	American Redstart	young forest
Setophaga cerulea	Cerulean Warbler	forest
Setophaga petechia	Yellow Warbler	open, edge
Setophaga pensylvanica	Chestnut-sided Warbler	early succession
Setophaga discolor	Prairie Warbler	early succession
Setophaga virens	Black-throated Green Warbler	forest
Cardellina canadensis	Canada Warbler	forest
Piranga olivacea	Scarlet Tanager	forest
Pheucticus ludovicianus	Rose-breasted Grosbeak	young forest
Icterus galbula	Baltimore Oriole	open
Short-distance migrants		
Aix sponsa	Wood Duck	wooded wetland, ponds
Buteo lineatus	Red-shouldered Hawk	forest
Colaptes auratus	Northern Flicker	open
Sayornis phoebe	Eastern Phoebe	open
Vireo griseus	White-eyed Vireo	early succession

Continued on next page

Scientific name	Common name	Habitat
Troglodytes aedon	House Wren	edge
Dumetella carolinensis	Gray Catbird	generalist
Toxostoma rufum	Brown Thrasher	early succession
Geothlypis trichas	Common Yellowthroat	early succession
Pipilo erythrophthalmus	Eastern Towhee	early succession
Spizella passerina	Chipping Sparrow	open
Spizella pusilla	Field Sparrow	early succession
Agelaius phoeniceus	Red-winged Blackbird	open
Quiscalus quiscula	Common Grackle	open
Molothrus ater	Brown-headed Cowbird	open
Permanent residents		
Colinus virginianus	Northern Bobwhite	early succession
Bonasa umbellus	Ruffed Grouse	young forest
Buteo jamaicensis	Red-tailed Hawk	open
Zenaida macroura	Mourning Dove	open
Strix varia	Barred Owl	forest
Melanerpes carolinus	Red-bellied Woodpecker	forest
Picoides pubescens	Downy Woodpecker	generalist
Picoides villosus	Hairy Woodpecker	forest
Dryocopus pileatus	Pileated Woodpecker	forest
Cyanocitta cristata	Blue Jay	generalist
Corvus brachyrhynchos	American Crow	open
Corvus ossifragus	Fish Crow	open
Poecile atricapillus	Black-capped Chickadee	generalist
Baeolophus bicolor	Tufted Titmouse	generalist

Continued on next page

Scientific name	Common name	Habitat
Sitta canadensis	Red-breasted Nuthatch	forest
Sitta carolinensis	White-breasted Nuthatch	forest
Certhia americana	Brown Creeper	forest
Thryothorus ludovicianus	Carolina Wren	edge
Sialia sialis	Eastern Bluebird	open
Turdus migratorius	American Robin	open
Mimus polyglottos	Northern Mockingbird	open
Sturnus vulgaris	European Starling	open
Bombycilla cedrorum	Cedar Waxwing	early succession
Melospiza melodia	Song Sparrow	early succession, open
Cardinalis cardinalis	Northern Cardinal	Open edge
Haemorhous mexicanus	House Finch	open
Spinus tristis	American Goldfinch	early succession

References

[1] R. M. DeGraaf and M Yamasaki. *New England wildlife: habitat, natural history, and distribution.* University Press of New England, Hanover, New Hampshire, 2001.
