# Supplementary material for: Estimating the Effects of Habitat and Biological Interactions in an Avian Community
Source: PLoS One. 2015 Aug 19;10(8):e0135987. doi: 10.1371/journal.pone.0135987 (PMC4543583; doi:10.1371/journal.pone.0135987)
Supplement: S2 Table — (PDF) [file pone.0135987.s005.pdf]

Estimating the effects of habitat and biological interactions in an avian community

Robert M. Dorazio, U.S. Geological Survey, Southeast Ecological Science Center, Gainesville, FL, USA

Edward F. Connor, Department of Biology, San Francisco State University, San Francisco, CA, USA

Robert A. Askins, Biology Department, Connecticut College, New London, CT USA

S2 Table: Summary of avian counts by species

Observed frequency (no. surveys) of number of birds detected per survey.

	Number of birds detected						
Species	0	1	2	3	4	5	6
Long-distance migrants							
Broad-winged Hawk	240	5	0	0	0	0	0
Yellow-billed Cuckoo	223	20	2	0	0	0	0
Black-billed Cuckoo	237	8	0	0	0	0	0
Eastern Whip-poor-will	243	2	0	0	0	0	0
Ruby-throated Hummingbird	244	1	0	0	0	0	0
Eastern Wood-Pewee	142	74	26	3	0	0	0
Acadian Flycatcher	239	6	0	0	0	0	0
Great Crested Flycatcher	135	87	23	0	0	0	0
Eastern Kingbird	244	0	1	0	0	0	0
Yellow-throated Vireo	228	17	0	0	0	0	0
Red-eyed Vireo	95	54	53	26	10	6	1
Blue-gray Gnatcatcher	233	10	2	0	0	0	0

Continued on next page

	Number of birds detected						
Species	0	1	2	3	4	5	6
Veery	103	78	44	17	3	0	0
Hermit Thrush	239	4	1	1	0	0	0
Wood Thrush	93	76	52	17	5	2	0
Ovenbird	58	55	55	33	24	13	7
Worm-eating Warbler	191	43	9	2	0	0	0
Louisiana Waterthrush	236	9	0	0	0	0	0
Blue-winged Warbler	227	16	2	0	0	0	0
Black-and-white Warbler	157	66	20	2	0	0	0
Hooded Warbler	220	17	6	2	0	0	0
American Redstart	225	17	2	1	0	0	0
Cerulean Warbler	239	6	0	0	0	0	0
Yellow Warbler	234	10	1	0	0	0	0
Chestnut-sided Warbler	241	4	0	0	0	0	0
Prairie Warbler	234	7	2	2	0	0	0
Black-throated Green Warbler	233	11	1	0	0	0	0
Canada Warbler	237	8	0	0	0	0	0
Scarlet Tanager	114	98	30	1	2	0	0
Rose-breasted Grosbeak	226	19	0	0	0	0	0
Baltimore Oriole	198	39	8	0	0	0	0
Short-distance migrants							
Wood Duck	244	1	0	0	0	0	0
Red-Shouldered Hawk	241	4	0	0	0	0	0
Northern Flicker	167	69	9	0	0	0	0
Eastern Phoebe	231	13	1	0	0	0	0

Continued on next page

Species	Number of birds detected 0	1	2	3	4	5	6
White-eyed Vireo	232	10	2	0	1	0	0
House Wren	176	36	23	8	2	0	0
Gray Catbird	182	38	15	8	2	0	0
Brown Thrasher	242	3	0	0	0	0	0
Common Yellowthroat	167	46	20	9	1	2	0
Eastern Towhee	66	85	53	31	9	1	0
Chipping Sparrow	244	1	0	0	0	0	0
Field Sparrow	244	1	0	0	0	0	0
Red-winged Blackbird	220	17	7	0	1	0	0
Common Grackle	225	16	3	1	0	0	0
Brown-headed Cowbird	178	64	3	0	0	0	0
Permanent residents							
Northern Bobwhite	221	16	8	0	0	0	0
Ruffed Grouse	240	4	1	0	0	0	0
Red-tailed Hawk	244	1	0	0	0	0	0
Mourning Dove	211	33	1	0	0	0	0
Barred Owl	243	2	0	0	0	0	0
Red-bellied Woodpecker	207	36	2	0	0	0	0
Downy Woodpecker	185	60	0	0	0	0	0
Hairy Woodpecker	213	31	1	0	0	0	0
Pileated Woodpecker	240	4	1	0	0	0	0
Blue Jay	79	114	44	7	1	0	0
American Crow	141	74	22	5	2	0	1
Fish Crow	242	2	1	0	0	0	0

Continued on next page

	Number of birds detected						
Species	0	1	2	3	4	5	6
Black-capped Chickadee	98	114	29	3	1	0	0
Tufted Titmouse	62	109	56	18	0	0	0
Red-breasted Nuthatch	244	1	0	0	0	0	0
White-breasted Nuthatch	162	80	3	0	0	0	0
Brown Creeper	223	20	2	0	0	0	0
Carolina Wren	223	21	1	0	0	0	0
Eastern Bluebird	243	2	0	0	0	0	0
American Robin	185	59	0	1	0	0	0
Northern Mockingbird	230	13	2	0	0	0	0
European Starling	230	10	4	0	1	0	0
Cedar Waxwing	244	1	0	0	0	0	0
Song Sparrow	222	19	3	1	0	0	0
Northern Cardinal	145	72	22	4	2	0	0
House Finch	244	0	1	0	0	0	0
American Goldfinch	237	8	0	0	0	0	0
