# Supplementary material for: Estimating the Effects of Habitat and Biological Interactions in an Avian Community
Source: PLoS One. 2015 Aug 19;10(8):e0135987. doi: 10.1371/journal.pone.0135987 (PMC4543583; doi:10.1371/journal.pone.0135987)
Supplement: S3 Table — (PDF) [file pone.0135987.s006.pdf]

Estimating the effects of habitat and biological interactions in an avian community

Robert M. Dorazio, U.S. Geological Survey, Southeast Ecological Science Center, Gainesville, FL, USA

Edward F. Connor, Department of Biology, San Francisco State University, San Francisco, CA, USA

Robert A. Askins, Biology Department, Connecticut College, New London, CT USA

S3: Species-specific estimates of abundance parameters

Estimates of parameters related to expected abundance of each species. Asterisks indicate species whose estimates were computed using the multispecies N-mixture model. LPC1–LPC2 and VPC1–VPC4 indicate effects of covariates (principal components of landscape and vegetation, respectively) on expected abundance. Tabulated covariate effects differed significantly from zero (using 5% significance level) with exception of effect of LPC2 on Hermit Thrush and effect of LPC1 on Prairie Warbler. Asymptotic standard error or posterior standard deviation is given in parenthesis.

Species	Intercept	LPC1	LPC2	VPC1	VPC2	VPC3	VPC4
Long-distance migrants							
Broad-winged Hawk	-4.72				1.20		
	(0.95)				(0.53)		
Yellow-billed Cuckoo	-2.10			0.39			
	(0.28)			(0.15)			
Black-billed Cuckoo	-1.42						
	(0.89)						
Eastern Wood-Pewee	-0.66	0.23					

Continued on next page

Species	Intercept	LPC1	LPC2	VPC1	VPC2	VPC3	VPC4
	(0.09)	(0.05)					
Acadian Flycatcher	-3.65						
	(0.41)						
Great Crested Flycatcher	-0.54						
	(0.09)						
Eastern Kingbird	-4.72						
	(0.72)						
Yellow-throated Vireo	-3.91	1.06					
	(0.60)	(0.25)					
Red-eyed Vireo*	0.05	0.23		-0.19	0.15		
	(0.07)	(0.04)		(0.04)	(0.04)		
Blue-gray Gnatcatcher	-3.44	0.70					
	(0.45)	(0.21)					
Veery	-0.07		-0.27		0.12		
	(0.07)		(0.09)		(0.05)		
Hermit Thrush	-7.42	2.65	-2.73		-0.49		
	(2.31)	(1.26)	(1.42)		(0.22)		
Wood Thrush	0.02	0.22					
	(0.07)	(0.04)					
Ovenbird	0.50	0.32	-0.22		0.10		
	(0.05)	(0.03)	(0.07)		(0.03)		
Worm-eating Warbler	-1.41	0.34					
	(0.14)	(0.08)					
Louisiana Waterthrush	-2.06						
	(0.50)						

Continued on next page

Species	Intercept	LPC1	LPC2	VPC1	VPC2	VPC3	VPC4
Blue-winged Warbler	-2.25	-0.40			0.55		
	(0.37)	(0.14)			(0.20)		
Black-and-white Warbler	-0.72			0.24			
	(0.10)			(0.07)			
Hooded Warbler*	-3.45	0.48					0.72
	(0.53)	(0.15)					(0.23)
American Redstart	-1.67						
	(0.26)						
Cerulean Warbler	-6.42	1.30			1.42		
	(1.53)	(0.47)			(0.56)		
Yellow Warbler	-5.85	-2.16	-1.54				
	(1.28)	(0.59)	(0.61)				
Chestnut-sided Warbler	-3.87						
	(0.51)						
Prairie Warbler*	-5.13	-0.41					
	(1.10)	(0.24)					
Black-throated Green Warbler	-4.36			-1.17			
	(0.69)			(0.24)			
Canada Warbler	-3.33						
	(0.36)						
Scarlet Tanager	-0.39	0.20					
	(0.08)	(0.05)					
Rose-breasted Grosbeak	-2.38						
	(0.24)						
Baltimore Oriole	-1.39						

Continued on next page

Species	Intercept	LPC1	LPC2	VPC1	VPC2	VPC3	VPC4
	(0.14)						
Short-distance migrants							
Northern Flicker	-0.90	-0.27					
	(0.13)	(0.06)					
Eastern Phoebe	-2.48						
	(0.30)						
White-eyed Vireo	-4.01	-1.03					
	(0.50)	(0.18)					
House Wren*	-1.36	-0.43					
	(0.19)	(0.07)					
Gray Catbird*	-1.43	-0.60					
	(0.17)	(0.07)					
Brown Thrasher	-3.84						
	(0.83)						
Common Yellowthroat	-1.11	-0.43		0.29	0.26		-0.28
	(0.13)	(0.06)		(0.07)	(0.08)		(0.08)
Eastern Towhee	0.23			0.28			
	(0.06)			(0.04)			
Field Sparrow	-5.41						
	(1.02)						
Red-winged Blackbird*	-3.79	-1.07	-0.84				
	(0.61)	(0.27)	(0.39)				
Common Grackle	-1.59	-0.39					
	(0.41)	(0.12)					
Brown-headed Cowbird	-0.78						

Continued on next page

Species	Intercept	LPC1	LPC2	VPC1	VPC2	VPC3	VPC4
	(0.16)						
<u>*Permanent residents*</u>							
Northern Bobwhite	-3.09	-0.91	-1.47		0.38		
	(0.39)	(0.20)	(0.39)		(0.15)		
Ruffed Grouse	-8.03	3.09	-3.56				
	(2.68)	(1.41)	(1.66)				
Mourning Dove	-1.74						
	(0.18)						
Red-bellied Woodpecker	-2.10	0.48			0.38		
	(0.25)	(0.11)			(0.14)		
Downy Woodpecker	-0.99						
	(0.16)						
Hairy Woodpecker	-1.88						
	(0.18)						
Pileated Woodpecker	-3.62						
	(0.42)						
Blue Jay	0.11						
	(0.07)						
American Crow*	-0.51	-0.19					
	(0.13)	(0.06)					
Fish Crow	-4.10						
	(0.5)						
Black-capped Chickadee	-0.14						
	(0.08)						
Tufted Titmouse	0.18						

Continued on next page

Species	Intercept	LPC1	LPC2	VPC1	VPC2	VPC3	VPC4
	(0.06)						
Red-breasted Nuthatch	-5.50						
	(1.00)						
White-breasted Nuthatch	-0.95						
	(0.11)						
Brown Creeper	-2.65				0.68	-0.52	
	(0.34)				(0.20)	(0.20)	
Carolina Wren	-2.74	-0.59					
	(0.31)	(0.14)					
American Robin	-0.93						
	(0.16)						
Northern Mockingbird	-2.96	-0.48					
	(0.32)	(0.15)					
European Starling*	-3.88	-0.85					
	(0.69)	(0.21)					
Cedar Waxwing	-5.41						
	(1.02)						
Song Sparrow	-3.55	-1.26	-0.94				
	(0.52)	(0.27)	(0.34)				
Northern Cardinal	-0.58	-0.31					
	(0.10)	(0.05)					
House Finch	-4.47						
	(0.84)						
American Goldfinch	-2.14						
	(0.89)						
