# Supplementary material for: Estimating the Effects of Habitat and Biological Interactions in an Avian Community
Source: PLoS One. 2015 Aug 19;10(8):e0135987. doi: 10.1371/journal.pone.0135987 (PMC4543583; doi:10.1371/journal.pone.0135987)
Supplement: S4 Table — (PDF) [file pone.0135987.s007.pdf]

Estimating the effects of habitat and biological interactions in an avian community

Robert M. Dorazio, U.S. Geological Survey, Southeast Ecological Science Center, Gainesville, FL, USA

Edward F. Connor, Department of Biology, San Francisco State University, San Francisco, CA, USA

Robert A. Askins, Biology Department, Connecticut College, New London, CT USA

S4 Table: Species-specific estimates of detection parameters

Estimates of parameters related to detection probability of each species. Asterisks indicate species whose estimates were computed using the multispecies N-mixture model. Tabulated day-of-year effects differed significantly from zero (using 5% significance level). Asymptotic standard error or posterior standard deviation is given in parenthesis.

Species	Intercept	Day of year
Long-distance migrants		
Broad-winged Hawk	-0.81	
	(0.62)	
Yellow-billed Cuckoo	-1.11	
	(0.32)	
Black-billed Cuckoo	-3.73	-1.05
	(1.06)	(0.43)
Eastern Wood-Pewee	0.57	
	(0.09)	
Acadian Flycatcher	0.04	

Continued on next page

Species	Intercept	Day of year
	(0.46)	
Great Crested Flycatcher	-0.02	
	(0.1)	
Eastern Kingbird	-0.18	
	(0.82)	
Yellow-throated Vireo	-0.34	
	(0.29)	
Red-eyed Vireo*	0.43	
	(0.06)	
Blue-gray Gnatcatcher	-0.18	
	(0.31)	
Veery	0.25	
	(0.07)	
Hermit Thrush	0.38	
	(0.37)	
Wood Thrush	0.41	
	(0.07)	
Ovenbird	0.67	
	(0.05)	
Worm-eating Warbler	0.02	
	(0.14)	
Louisiana Waterthrush	-2.76	-1.16
	(0.74)	(0.44)
Blue-winged Warbler	-1.85	-1.47
	(0.40)	(0.35)

Continued on next page

Species	Intercept	Day of year
Black-and-white Warbler	-0.34	
	(0.11)	
Hooded Warbler*	0.32	
	(0.18)	
American Redstart	-1.63	-1.06
	(0.38)	(0.27)
Cerulean Warbler	-0.63	-2.65
	(0.58)	(0.91)
Yellow Warbler	-1.21	
	(0.47)	
Chestnut-sided Warbler	2.13	-4.07
	(1.53)	(1.92)
Prairie Warbler*	-0.03	
	(0.27)	
Black-throated Green Warbler	-0.64	
	(0.36)	
Canada Warbler	-0.18	
	(0.41)	
Scarlet Tanager	0.20	
	(0.09)	
Rose-breasted Grosbeak	-0.56	
	(0.29)	
Baltimore Oriole	-0.22	
	(0.16)	
Short-distance migrants		

Continued on next page

Species	Intercept	Day of year
Northern Flicker	-0.77	
	(0.15)	
Eastern Phoebe	-0.94	
	(0.38)	
White-eyed Vireo	0.32	
	(0.26)	
House Wren*	0.81	
	(0.11)	
Gray Catbird*	0.07	
	(0.11)	
Brown Thrasher	-1.45	
	(1.03)	
Common Yellowthroat	0.12	
	(0.10)	
Eastern Towhee	0.25	
	(0.06)	
Field Sparrow	-0.18	
	(1.16)	
Red-winged Blackbird*	0.01	
	(0.19)	
Common Grackle	-1.99	
	(0.45)	
Brown-headed Cowbird	-1.28	-0.41
	(0.20)	(0.14)
Permanent residents		

Continued on next page

Species	Intercept	Day of year
Northern Bobwhite	0.30	
	(0.19)	
Ruffed Grouse	-1.01	
	(0.61)	
Mourning Dove	-0.65	
	(0.22)	
Red-bellied Woodpecker	-0.67	
	(0.21)	
Downy Woodpecker	-1.17	
	(0.20)	
Hairy Woodpecker	-0.26	0.45
	(0.21)	(0.18)
Pileated Woodpecker	-0.18	
	(0.48)	
Blue Jay	-0.58	
	(0.09)	
American Crow*	-0.58	0.26
	(0.11)	(0.11)
Fish Crow	0.75	
	(0.56)	
Black-capped Chickadee	-0.36	0.30
	(0.09)	(0.09)
Tufted Titmouse	-0.03	
	(0.07)	
Red-breasted Nuthatch	1.08	

Continued on next page

Species	Intercept	Day of year
	(1.18)	
White-breasted Nuthatch	-0.22	
	(0.13)	
Brown Creeper	-0.76	
	(0.28)	
Carolina Wren	-0.36	
	(0.25)	
American Robin	-1.22	0.40
	(0.20)	(0.16)
Northern Mockingbird	0.13	
	(0.27)	
European Starling*	-0.89	
	(0.30)	
Cedar Waxwing	-0.18	
	(1.16)	
Song Sparrow	-0.18	
	(0.22)	
Northern Cardinal	-0.48	
	(0.11)	
House Finch	-1.01	
	(1.06)	
American Goldfinch	-2.47	
	(1.01)	
